# L-Carnitine’s Effect on the Biomarkers of Metabolic Syndrome: A Systematic Review and Meta-Analysis of Randomized Controlled Trials

**DOI:** 10.3390/nu12092795

**Published:** 2020-09-12

**Authors:** Munji Choi, Seongmin Park, Myoungsook Lee

**Affiliations:** 1 Department of Food and Nutrition & Research Institute of Obesity Sciences, Sungshin Women’s University, Seoul 01133, Korea; mjchoi@nfsi.or.kr (M.C.); kpsm3003@korea.kr (S.P.); 2 Center for Food and Drug Analysis, Gyeongin Regional Korea Food and Drug Administration, Incheon 402835, Korea

**Keywords:** L-carnitine, metabolic syndrome, waist circumference, blood pressure, fasting blood sugar, triglyceride, HDL-cholesterol

## Abstract

A systematic review and meta-analysis of randomized controlled trials (RCTs) was carried out to assess L-carnitine supplements’ influence on the biomarkers of metabolic syndrome (MetSyn). PubMed, EMBASE, Cochrane library, and CINAHL were used to collect RCT studies published prior to February 2020. RCT studies were included if they had at least one of the following biomarker outcome measurements: waist circumference (WC), blood pressure (BP), fasting blood sugar (FBS), triglyceride (TG), or high density lipoprotein-cholesterol (HDLc). Nine of twenty studies with adequate methodological quality were included in this meta-analysis. The dose of L-carnitine supplementation administered varied between 0.75 and 3 g/day for durations of 8–24 weeks. L-carnitine supplementation significantly reduced WC and systolic BP (SBP), with no significant effects on FBS, TG, and HDLc. We found that L-carnitine supplementation at a dose of more than 1 g/d significantly reduced FBS and TG and increased HDLc. In conclusion, L-carnitine supplementation is correlated with a significant reduction of WC and BP. A dose of 1–3 g/d could improve the biomarkers of MetSyn by reducing FBS and TG and increasing HDLc.

## 1. Introduction

Metabolic syndrome (MetSyn) is a significant threat to public health, resulting in increased mortality, disability, and medical expenses, and it has increased in prevalence over the past decades. MetSyn is a cluster of risk factors associated with cardiovascular disease and diabetes development, first described by Reaven in 1988 [1]. These factors include insulin resistance, type 2 diabetes or impaired glucose tolerance, hypertension, dyslipidemia, and abdominal obesity [2].

Carnitine exists in two forms, D-carnitine and L-carnitine, but only L-carnitine has biological action, while D-carnitine inhibits the action of L-type carnitine [3]. L-carnitine is a non-protein amino acid, the primary sources of which in humans include both diet and endogenous synthesis. It is widely found in animal foods such as meat, fish, milk, and dairy products and is mainly synthesized in the liver and kidneys from lysine and methionine [4]. L-carnitine plays a vital role in lipid metabolism by transporting long-chain fatty acids into the mitochondria, thereby stimulating beta-oxidation. 

Regarding the two effects of L-carnitine on glucose and lipid metabolism, it may help metabolic disorders such as type 2 diabetes and hypertriglyceridemia. L-carnitine is a popular supplement for weight loss and fat burning purposes. A meta-analysis showed that L-carnitine supplementation reduced body weight, body mass index, and fat mass [5,6]. L-carnitine supplementation can affect BP levels by reducing interactions with the nitric oxide pathway and insulin resistance [7]. Askarpour et al. [8] indicated that L-carnitine supplementation at a dose of ≥2 g/day decreases diastolic blood pressure (DBP) without affecting systolic BP (SBP) levels. L-carnitine supplementation at a dose of 2–3 g/day was associated with improved fasting blood sugar (FBS) and insulin resistance by increasing carbohydrate oxidation and reducing fatty acid oxidation [9,10]. L-carnitine is a critical element in the beta-oxidation of fatty acids and reduces free fatty acid availability for triglyceride (TG) synthesis. Malaguarnera et al. [11] reported that L-carnitine significantly decreased TG concentrations, whereas it increased HDLc concentrations.

To date, no meta-analysis on the use of L-carnitine supplements has been conducted to improve the biomarkers of MetSyn. It is crucial to systematically review the existing evidence regarding L-carnitine intake in improving the biomarkers of MetSyn. This study is the first to investigate the effect of L-carnitine supplementation on the biomarkers of MetSyn.

## 2. Materials and Methods

### 2.1. Search Strategy and Data Collection

The following electronic databases were searched for articles: PubMed, EMBASE, the Cochrane Library, and CINAHL (EBSCO). The search time frame ranged from the date of inception of each database until February 2020. The search terms used were as follows: intervention (“carnitine” OR “L-carnitine” OR “L-carnitine tartrate”), outcomes (“metabolic syndrome” OR “syndrome X” OR “Metabolic Syndrome X” OR “Insulin Resistance Syndrome X” OR “Metabolic X Syndrome” OR “Reaven Syndrome X” OR “Metabolic Cardiovascular Syndrome” OR “HDL Lipoprotein” OR “High-Density Lipoprotein” OR “HDL” OR “HDL-c” OR “HDL cholesterol” OR “cholesterol” OR “hypertension” OR “High Blood Pressure” OR “Blood Pressure” OR “Triacylglycerol” OR “Triglyceride” OR “Fasting Blood Sugar” OR “Fasting Blood glucose” OR “FBS” OR “Waist Circumference” OR “WC”), and study design (“intervention” OR “trial” OR “randomized” OR “randomized” OR “random” OR “randomly” OR “placebo” OR “RCT”). This study was restricted to articles published in the English language.

### 2.2. Inclusion and Exclusion Criteria

The inclusion criteria for this meta-analysis were (a) population: involving men and women aged over 18 years; (b) intervention: L-carnitine supplement (without other drugs) intervention for at least two weeks; (c) control: control group not receiving the intervention; (d) outcomes: changes in waist circumference (WC), BP, FBS, TG, or HDLc; and (e) study design: randomized controlled trial (RCT). This review’s exclusion criteria were as follows: nonhuman subjects; non-intervention; intervention for carnitine deficiency; carnitine combined with other medications; non-RCT studies, including cohort, case–control, cross-sectional, reviews, and commentaries; discordance in the outcome of MetSyn; and non-English publications.

Two reviewers (MC and SP) independently selected articles by applying the inclusion and exclusion criteria to identify relevant studies. Any trials that were not excluded based on title and abstract were reviewed in full-text by both reviewers. Any discrepancies between reviewers at each step were resolved through discussion until consensus was reached. Disagreements between reviewers were resolved by a third reviewer (ML).

### 2.3. Data Extraction and Quality Assessment

Data extraction was completed by one reviewer (MC) and was checked for accuracy by another reviewer (SP). Data were extracted on the study information (author’s surname, country location, and publication year), characteristics of the subjects (total sample size), and details of the trial (duration and dose of intervention, and biomarkers of MetSyn).

The quality assessment was done using the Cochrane Risk of Bias (ROB) tool. [12] For assessing the ROB in RCTs, each component was categorized as having high, low, or unclear ROB; the components were random sequence generation, allocation concealment, blinding of participants and personnel, blinding of outcome assessment, incomplete outcome data, selective outcome reporting, and other sources of bias. Two reviewers (MC and SP) determined each article’s quality, and a consensus was reached on the ROB score through discussion. A third reviewer (ML) resolved disagreements about the risk of bias.

### 2.4. Data Analysis

The mean differences (MDs) between the baseline and final value of the factors under study were extracted from the studies. Meta-analysis was conducted using RevMan software (version 5.3; Review Manager (RevMan), Nordic Cochrane Centre, Copenhagen, Denmark). The I^2^ statistic was used to assess for statistical heterogeneity amongst studies. A fixed-effects model was used if there was no significant heterogeneity of the data, I^2^ ≤ 50%; I^2^ > 50% with *p* ≤ 0.1 indicated statistical heterogeneity, and a random-effects model was then adopted [12]. Sub-group analysis was also conducted via the same methods.

When the standard deviation (SD) for mean differences was not reported, it was calculated by the following formula: SD = square root((SD baseline)^2^ + (SD final)^2^ − (2R × SD baseline × SD final)), assuming a correlation coefficient (R) of 0.5 [13]. Effect sizes were expressed as the MD and a 95% confidence interval (CI). Funnel plots were used to assess the publication bias.

## 3. Results

### 3.1. Study Selection

**Figure 1:**
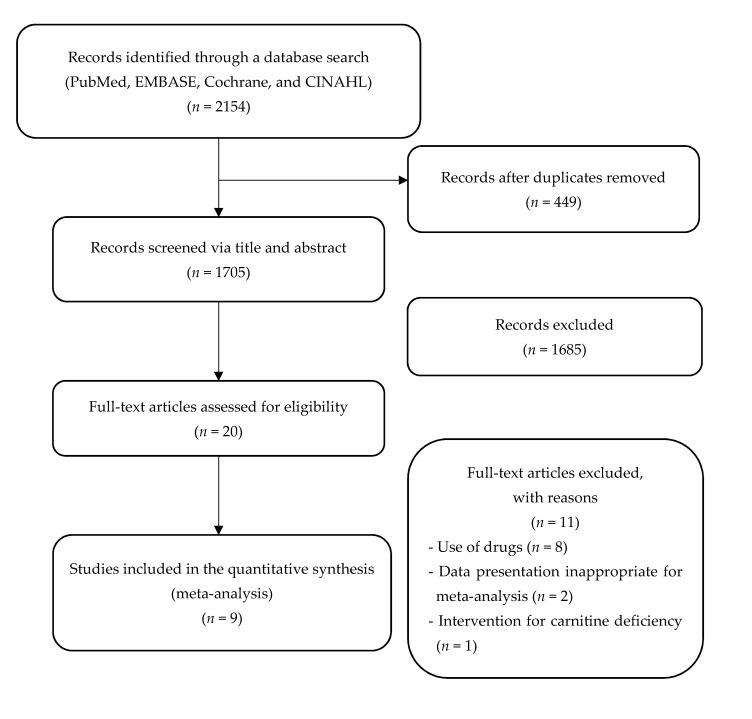
Flow chart of the study selection process.

The search identified 2154 articles, and following the removal of duplicates, the titles and abstracts of 1705 articles were screened. Most of the articles (*n* = 1685) were excluded after reading the titles or abstracts, since they were not relevant. After assessing the full text of 20 potentially related articles, 9 articles were included in our analysis. The most important reasons for exclusion were as follows: eight articles reported L-carnitine administered in combination with other components (or drugs), two articles were presented inappropriately for a meta-analysis, and one article was an intervention study on carnitine deficiency. Figure 1 shows the details of the study identification and selection process.

### 3.2. Characteristics of the Included RCT Studies

The review included 508 participants from nine studies in meta-analyses, and detailed information of the included studies is summarized in Table 1. Five of the studies were conducted in Iran [14,15,16,17,18], and two in Italy [11,19]. The other trials were conducted in Japan [20] and China [21]. The studies were performed in subjects with diabetes mellitus [11,14,21], non-alcoholic steatohepatitis [14,15,19], and knee osteoarthritis [16,17] and in those undergoing hemodialysis [18,20]. The mean age of the subjects was at least 41.6 years in each study. The sample size was from 18 to 81. The duration of treatment in the studies was between 8 and 24 weeks, and 12 weeks was the most common duration used for interventions. Six RCTs used L-carnitine alone [14,16,17,18,20,21]; one RCT used L-carnitine in combination with calorie restriction [11]; and two RCTs used a combination of L-carnitine, calorie restriction, and exercise [15,19]. The daily dose of L-carnitine used for supplementation ranged from 0.75 to 3 g/day, and 2 g/day was the most common dose used for intervention. Two RCTs showed changes in WC, two in BP, five in FBS, six in TG, and five in HDLc.

### 3.3. Risk of Bias in the Included RCT Studies

**Figure 2:**
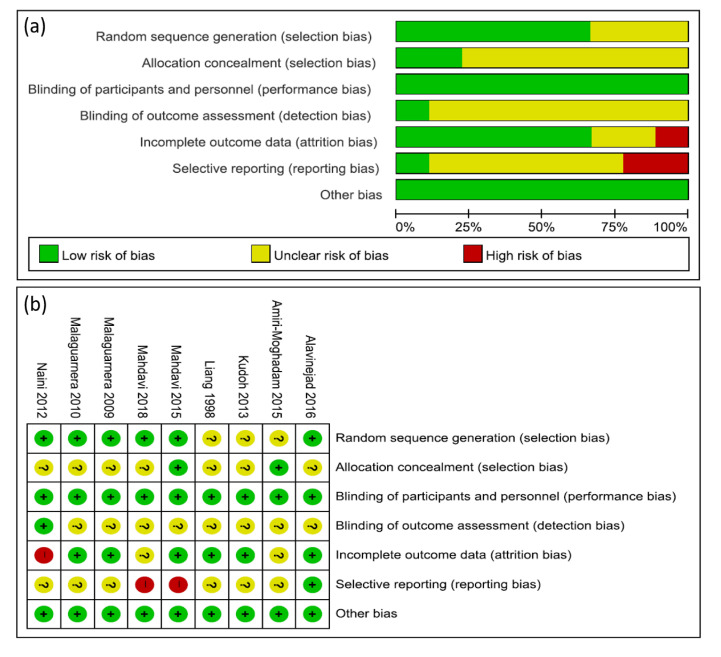
Risk of bias graph (**a**) and bias summary (**b**). Risk of bias levels: low (green or “+”), Unclear (yellow or “?”), High (red or “-“)

The quality of each of the nine RCTs was evaluated with regard to seven aspects using the ROB scale in the Cochrane Handbook for Systematic Reviews of Interventions of the Cochrane Collaboration. Although all the included trials reported “randomly allocating” participants, only seven studies reported generating random numbers by using a computer-generated randomization schedule, a random number table, envelopes, random block, or centralized randomization [11,14,17,18,19,20,21]. Two studies described allocation concealment [15,16]. Eight studies reported the dropout rate [11,14,16,17,18,19,20,21], but the remaining study was unclear about the dropout rate [15]. Three studies provided information about the trial registry [14,16,17]. Reporting bias was assessed by judging the consistency between results in the method section of the publication and the protocol. All other trials provided no information on the trial registry. This was assessed as an unclear risk of bias. For other biases, all studies were assessed as having a low risk for baseline comparability (Figure 2).

### 3.4. Effects of L-Carnitine Supplementation on Biomarkers of MetSyn

**Figure 3:**
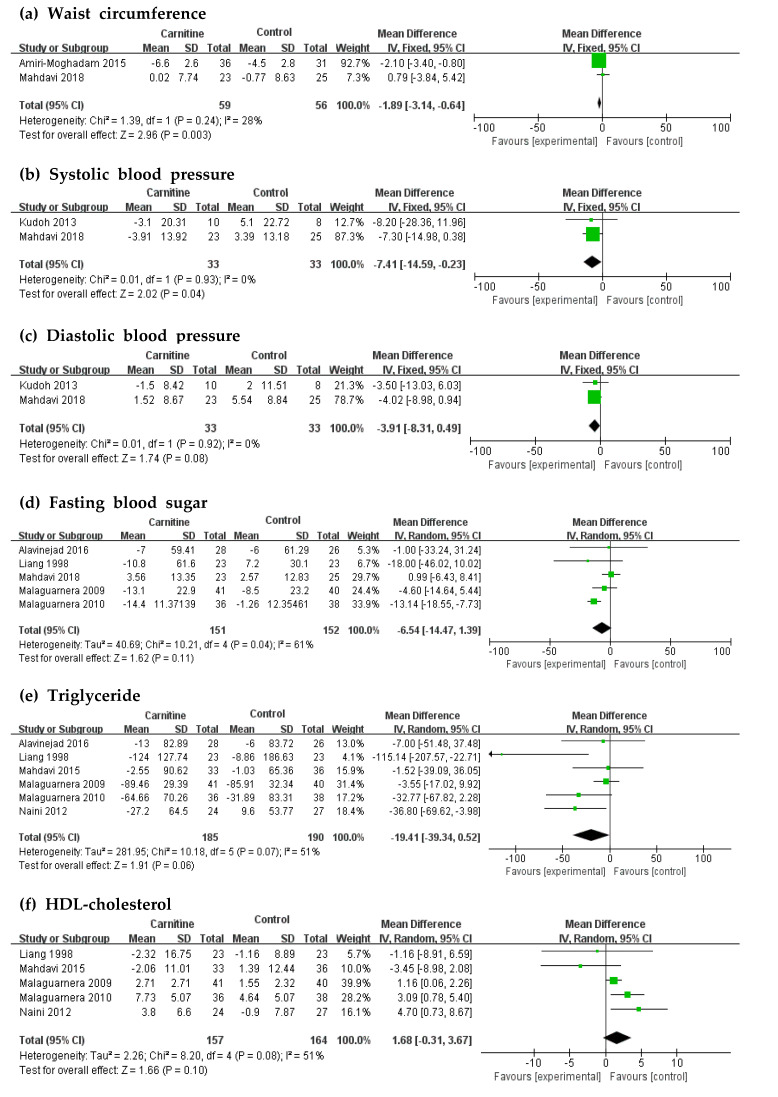
Forest plots of waist circumference (**a**), systolic blood pressure (**b**), diastolic blood pressure (**c**), fasting blood sugar (**d**), triglyceride (**e**), and HDL-cholesterol (**f**). **Abbreviations:** SD: standard deviation, IV: inverse variance, CI: confidence interval; HDL-Cholesterol: High density lipoprotein-cholesterol.

Forest plots of the biomarkers of MetSyn are shown in Figure 3. Two of the nine RCTs, totaling 155 participants, reported data on WC (Figure 3a). When compared with the control group, L-carnitine showed a significant WC decreasing effect of −1.89 cm (95% CI: −3.14 to −0.64, *p* = 0.003). Two of the nine RCTs, totaling 66 participants, reported data on SBP (Figure 3b). L-carnitine supplementation was related to a mean decrease in SBP of –7.41 mmHg (95% CI: −14.59 to −0.23, *p* = 0.04). The effect of L-carnitine on DBP was reported by two RCTs, but did not reveal a significant decrease in comparison with the control group (Figure 3c). Similarly, FBS, TG, and HDLc with L-carnitine intervention presented no significant difference between the intervention and control groups (Figure 3d–f). 

### 3.5. Subgroup Analysis and Heterogeneity

Table 2 shows the data from subgroup analysis by the population’s baseline status. The level of FBS in hyperglycemic patients (FBS at baseline of ≥ 100 mg/dL) was decreased by 10.74 mg/dL (95% CI: −15.90 to −5.58, I^2^ = 0%, *p* < 0.0001, four RCTs, *n* = 321). Additionally, in hypo-HDL cholesterolemia patients (HDLc at baseline of <40 mg/dL), meta-analysis of two studies revealed a significant HDLc-increasing effect of L-carnitine when compared with the control groups (subtotal MD = 3.50, 95% CI: 1.50 to 5.47, I^2^ = 0%, *p* = 0.0006, two RCTs, *n* = 125).

In the subgroup analysis by country (Table 3), we found significantly decreased FBS (subtotal MD = −9.96, 95% CI: −18.05 to −1.87, I^2^ = 54%, *p* = 0.02) and increased HDLc in the Italian group (subtotal MD = 1.85, 95% CI: 0.04 to 3.66, I^2^ = 54%, *p* = 0.05).

Table 4 shows the data from RCTs with high doses (more than 1 g per day) and low doses (less than 1 g per day) of L-carnitine. The levels of FBS and TG in the high-dose groups were significantly decreased (FBS, −11.41 mg/dL, −16.10 to −6.72 mg/dL; TG, −29.85 mg/dL, −60.08 to 0.38 mg/dL). HDLc levels were significantly increased by 1.66 mg/dL in the high-dose groups (95% CI: 0.70, 2.61; I^2^ = 40%; *p* = 0.0007; four RCTs; *n* = 252). Non-significant results were found for the levels of FBS, TG, and HDLc in the low-dose groups.

## 4. Discussion

In this study, we aimed to present an overall result of how L-carnitine supplementation affects the biomarker components of MetSyn using a meta-analysis. This meta-analysis included nine random, placebo-controlled trials comprising 508 participants. Most of the included trials had relatively adequate methodological quality. L-carnitine supplementation showed significant lowering effects on WC and SBP, but did not show significant effects in terms of FBS, TG, and HDLc when compared with the placebo group. In agreement with our findings, L-carnitine supplementation was found to significantly reduce body weight and body mass index in a meta-analysis that confirmed the effect on weight loss of L-carnitine supplementation [5]. For BP, this review found significant reductions in SBP, but not in DBP, following L-carnitine supplementation. In a recent meta-analysis of RCTs, L-carnitine supplementation was found to reduce DBP without changing SBP [8]. Ruggenenti et al. [22] reported that 2 g per day of oral acetyl-L-carnitine, an L-carnitine ester, effectively decreased SBP without affecting DBP in nondiabetic hypertensive participants with a high cardiovascular risk profile. The benefits of L-carnitine supplementation in terms of BP levels are in part due to improved insulin sensitivity and glucose control [23]. Hyperinsulinemia and insulin resistance are risk factors for increased BP [24,25]. According to the “energy starvation” hypothesis, carnitine can improve cardiomyocytes’ energy metabolism, thereby improving the mechanical efficiency and function of cardiac fibroblasts and regulating BP [26,27]. In contrast to other studies that reported a decrease in FBS in type 2 diabetes mellitus populations [9], this review did not find any effect on FBS. Vidal-Casariego et al. [9] evaluated the metabolic effects of L-carnitine administration in type 2 diabetes mellitus patients via systematic review and meta-analysis. A meta-analysis of four studies demonstrated that oral L-carnitine lowered FBS. Two of them were studies involving L-carnitine supplementation and drugs taken together, and one of them focused on acetyl-L-carnitine. In the present study, the three studies mentioned above were excluded; thus, it seems that there was a difference in the results of these studies. When subgroup analysis was performed by the population’s baseline status and by country, the FBS-lowering effect of L-carnitine supplementation was confirmed in an Italian population and in a population with baseline FBS of ≥100 mg/dL. L-carnitine supplementation can improve glucose metabolism via several mechanisms. First, the enhancement of mitochondrial oxidation of accumulated long-chain acyl-CoA produces insulin resistance in muscle and the heart. Second, it induces changes in glycolytic and gluconeogenic enzymes. Third, it modifies the expression of genes associated with the insulin-signaling cascade. Finally, it improves glucose utilization by the heart [28].

Our findings are consistent with those of a previous meta-analysis study that suggested that carnitine does not significantly decrease TG in type 2 diabetes mellitus patients [9], hemodialysis patients [29,30], and other populations [31]. Of the six RCTs used in the present meta-analysis, four showed carnitine to be effective [11,18,19,21], and two showed it to be ineffective [14,16]. In the two latter RCTs, the L-carnitine supplementation dose was 0.7 g. In our subgroup analysis by the population’s baseline status (TG ≥ 150 mg/dL) and by country, there was no significant decrease in TG. Theoretically, it is known that L-carnitine may increase the mitochondrial transport of fatty acids and reduce fatty acid availability for lipid synthesis [32]. Based on both previous research results and the current research results, the functions of L-carnitine taken in the form of supplements and those of synthesized carnitine may be different.

Some studies reported that HDLc was unchanged by supplementation of L-carnitine [9,31]. Only one of the five RCTs used in the meta-analysis of HDLc showed a significant improvement effect when compared to the control group. HDLc is known to be increased by exercise, and in one study, L-carnitine supplementation and exercise programs proved to be effective. In our subgroup analysis by population’s baseline status and by country, the HDLc-increasing effect of L-carnitine supplementation was confirmed in an Italian population and in a population with baseline HDLc of <40 mg/dL.

Furthermore, in the subgroup analysis based on L-carnitine dose, when the treatment dose was more than 1 g/d, FBS, TG, and HDLc were significantly improved. However, when the L-carnitine dose was under 1 g/d, there was no change. These results suggest that at least 1 g/d of L-carnitine should be consumed to improve MetSyn biomarkers.

Supplementation of L-carnitine at 5 g/day or more has been reported to have some adverse events, including diarrhea [33] and the production of trimethylamine-N-oxide, linked to an increased risk of atherosclerosis [34]. Ultimately, 2–3 g/day of supplemented L-carnitine is recommended. Values of the estimated average requirement, adequate intake, or upper levels for L-carnitine could be proposed at the 2020 Korean Dietary Reference Intakes (KDRI) revision by the KDRI committee. However, we will have to postpone making a decision until 2025, because of the lack of scientific evidence, such as daily intakes in Korean, dose–response results, or a comparison of intakes and body stores. The National Health & Medical Research Council have not considered a DRI for L-carnitine, because it is a non-essential nutrient [35]. Since 2 g/day of L-carnitine has been reported as “the upper level for supplements” in risk assessment studies, this human tolerance level of L-carnitine will be recommended [36,37].

There are several limitations to the current study. Firstly, we investigated only the association between synthetic L-carnitine supplements, without considering sources from food, and the biomarkers of MetSyn. Secondly, this meta-analysis was not conducted on patients diagnosed with MetSyn. Since the prevalence of MetSyn varies depending on the selected criteria of MetSyn, it is more suitable to use the biomarkers (criteria) for diagnosing MetSyn rather than subjects diagnosed with MetSyn. Thirdly, the RCT terms of L-carnitine supplementation varied among the studies. Fourth, some of the articles included in the analysis of WC and BP were conducted only on female patients. Lastly, to analyze L-carnitine supplementation’s clinical effects on some biomarkers, few eligible RCTs and few countries were included in the meta-analysis.

## 5. Conclusions

L-carnitine supplementation is correlated with a significant reduction in WC and BP. Additionally, L-carnitine supplementation at a dose of 1–3 g/d could improve MetSyn by reducing FBS and TG and increasing HDLc.

## Figures and Tables

**Table 1:** Characteristics of the included studies.

Study		Population				Intervention		Outcomes of MetSyn
Author (Country)	Year	Case	Mean Age (Int/Cont)	Sex (M/F)	Sample Size (Int/Cont)	Duration (Weeks)	Oral Dose (g/day)	
Alavinejad et al. [14] (Iran)	2016	T2DM and NASH	60.0/59.0	38/16	28/26	12	0.75	FBS, TG
Moghadam et al. [15] (Iran)	2015	NASH	41.6/45.3	23/44	36/31	12 (+CR, EX)	2	WC
Kudoh et al. [20] (Japan)	2013	Under hemo-dialysis	65.9/67.8	8/10	10/8	12	0.9	SBP, DBP
Liang et al. [21] (China)	1998	T2DM	59.4/57.9	16/30	23/23	12	3	FBS, TG, HDLc
Mahdavi et al. [16] (Iran)	2015	Overweight or obese women with knee OA	51.63/52.44	0/69	33/36	8	0.75	TG, HDLc
Mahdavi et al. [17] (Iran)	2018	Obese women with knee OA	51.56/52.60	0/48	23/25	8	0.75	WC, FBS, SBP, DBP
Malaguarnera et al. [11] (Italy)	2009	T2DM	49.0/48.0	58/23	41/40	12 (+CR)	2	FBS, TG, HDLc
Malaguarnera et al. [19] (Italy)	2010	NASH	47.9/47.8	40/34	36/38	24 (+CR, EX)	2	FBS, TG, HDLc
Naini et al. [18] (Iran)	2012	Under hemo-dialysis	53.9/51.8	26/25	24/27	16	1	TG, HDLc

**Abbreviations**: int: intervention, cont: control, M: male, F: female, T2DM: type 2 diabetes mellitus, NASH: non-alcoholic fatty liver, OA: osteoarthritis, CR: calorie restriction, EX: exercise, FBS: fasting blood sugar, TG: triglyceride, WC: waist circumference, SBP: systolic blood pressure, DBP: diastolic blood pressure, HDLc: High density lipoprotein-cholesterol.

**Table 2:** Subgroup analysis by baseline status.

	A Quantitative Synthesis of Data				Heterogeneity of Data	
Outcome	No. of RCTs	MD	95% CI	*p*-Value	I^2^	*p*-Value
FBS						
Baseline FBS ≥ 100 mg/dL [11,14,19,21]	4	−10.74	−15.90, −5.58	<0.0001	0%	0.46
Baseline FBS < 100 mg/dL [17]	1	0.99	−6.43, 8.41	0.79	-	-
TG						
Baseline TG ≥ 150 mg/dL [11,14,19,21]	4	−21.48	−50.56, 7.59	0.15	60%	0.06
Baseline TG < 150 mg/dL [16,18]	2	−20.39	−54.88, 14.09	0.25	48%	0.17
HDL-C						
Baseline HDLc < 40 mg/dL [18,19]	2	3.5	1.50, 5.49	0.0006	0%	0.49
Baseline HDLc ≥ 40 mg/dL [11,16,21]	3	0.94	−0.12, 2.01	0.08	30%	0.24

**Abbreviations**: RCT: randomized controlled trials, MD: mean differences, CI: confidence interval, FBS: fasting blood sugar, TG: triglyceride, HDL-C: High density lipoprotein-cholesterol.

**Table 3:** Subgroup analysis by country.

	A Quantitative Synthesis of Data				Heterogeneity of Data	
Outcome	No. of RCTs	MD	95% CI	*p*-Value	I^2^	*p*-Value
FBS						
Iran [14,17]	2	0.89	−6.34, 8.12	0.81	0	0.91
Italy [11,19]	2	−9.96	−18.05, −1.87	0.02	54	0.14
China [21]	1	−18.00	−46.02, 10.02	0.21	-	-
TG						
Iran [14,16,18]	3	−17.76	−40.69, 5.18	0.13	10	0.33
Italy [11,19]	2	−13.50	−40.63, 13.64	0.33	57	0.13
China [21]	1	−115.14	−207.57, −22.71	0.01	-	-
HDL-C						
Iran [16,18]	2	0.86	−7.11, 8.84	0.83	82	0.02
Italy [11,19]	2	1.85	0.04, 3.66	0.05	54	0.14
China [21]	1	−1.16	−8.91, 6.59	0.77	-	-

**Abbreviations**: RCT: randomized controlled trials, MD: mean differences, CI: confidence interval, FBS: fasting blood sugar, TG: triglyceride, HDL-C: High density lipoprotein-cholesterol.

**Table 4:** Subgroup analysis by L-carnitine dose.

	A Quantitative Synthesis of Data				Heterogeneity of Data	
Outcome	No. of RCTs	MD	95% CI	*p*-Value	I^2^	*p*-Value
FBS						
≥1 g/d L-carnitine [11,19,21]	3	−11.41	−16.10, −6.72	<0.0001	16%	0.31
<1 g/d L-carnitine [14,17]	2	0.89	−6.34, 8.12	0.81	0%	0.91
TG						
≥1 g/d L-carnitine [11,19,21]	3	−29.85	−60.08, 0.38	0.05	69%	0.02
<1 g/d L-carnitine [14,16]	2	−3.8	−32.50, 24.90	0.08	0%	0.85
HDL-C						
≥1 g/d L-carnitine [11,18,19,21]	4	1.66	0.70, 2.61	0.0007	40%	0.17
<1 g/d L-carnitine [16]	1	−3.45	−8.98, 2.08	1.22	-	-

**Abbreviations**: RCT: randomized controlled trials, MD: mean differences, CI: confidence interval, FBS: fasting blood sugar, TG: triglyceride, HDL-C: High density lipoprotein-cholesterol.
